# Gregarines modulate insect responses to sublethal insecticide residues

**DOI:** 10.1007/s00442-021-05086-4

**Published:** 2021-12-01

**Authors:** Marina Wolz, Alia Schrader, Eileen Whitelaw, Caroline Müller

**Affiliations:** Department of Chemical Ecology, Bielefeld University, Universitätsstr. 24, 33615 Bielefeld, Germany

**Keywords:** Combined challenges, Fitness, Gregarines, Leaf beetle, Pyrethroid

## Abstract

Throughout their lifetime, insects face multiple environmental challenges that influence their performance. Gregarines are prevalent endoparasites in most invertebrates that affect the fitness of their hosts, but are often overlooked in ecological studies. Next to such biotic factors, a current common challenge is anthropogenic pollution with pesticides, which causes a major threat to non-target organisms that are readily exposed to lethal or sublethal concentrations. In a laboratory study, we investigated whether the presence of gregarines modulates the food consumption and life history traits of a (non-target) leaf beetle species, *Phaedon cochleariae*, in response to sublethal insecticide exposure. We show that the larval food consumption of the herbivore was neither affected by gregarine infection nor sublethal insecticide exposure. Nevertheless, infection with gregarines led to a delayed development, while insecticide exposure resulted in a lower body mass of adult males and a reduced reproduction of females. Individuals exposed to both challenges suffered most, as they had the lowest survival probability. This indicates detrimental effects on the population dynamics of non-target insects infected with naturally occurring gregarines that face additional stress from agrochemical pollution. Moreover, we found that the infection load with gregarines was higher in individuals exposed to sublethal insecticide concentrations compared to unexposed individuals. To counteract the global decline of insects, the potential of natural parasite infections in modulating insect responses to anthropogenic and non-anthropogenic environmental factors should be considered in ecological risk assessment.

**Supplementary Information:**

The online version contains supplementary material available at 10.1007/s00442-021-05086-4.

## Introduction

Throughout their lifetime, organisms have to cope with a large number of environmental challenges including parasite infections. In invertebrates, gregarines (Apicomplexa) are probably one of the most common endosymbionts, often considered as endoparasites (Locklin and Vodopich 2010; Logan et al. 2012). They can lead to a delayed development, higher mortality, and reduced reproduction of their hosts (Zuk 1987a; Córdoba-Aguilar et al. 2003). Nutrient deprivation induced by the gregarines may be causing some of these negative effects (Gigliolli et al. 2016). However, gregarine infections can also be benign for their hosts (Harry 1967; Kim et al. 2014) or even beneficial, for example, by accelerating the development or lowering predation (Alarcón et al. 2017; Soghigian et al. 2017). The relationship between gregarines and their hosts, i.e., whether parasitic, neutral, or mutualistic, seems to be highly species-specific and depending on the gregarine load (Zuk 1987a; Field and Michiels 2005; Rueckert et al. 2019). Furthermore, the fitness of the host may alter the outcome of the interaction, for example, under suboptimal environmental conditions. Little is known about such interactive effects between gregarines and other environmental factors that affect insect life histories.

One of these factors is anthropogenic stress such as agrochemical pollutants that have a huge impact on organisms and ecosystems (Köhler and Triebskorn 2013; Guedes et al. 2016; Müller 2018). For example, insecticide residues are known to impact the behaviour, physiological processes and fitness-related parameters of various invertebrates that are not targeted on purpose by the pesticides (Stark and Banks 2003; Desneux et al. 2007). The specific effects depend on the mode of action of the insecticide, the concentration the organism is facing, and the invertebrate species itself (Guedes et al. 2017; Müller 2018). Pyrethroids are a widely used class of insecticides, which act as neurotoxins by retarding the closing of sodium channels (Soderlund et al. 2002). In that way, sublethal concentrations of pyrethroids pose a threat to target pests but also to non-target arthropods, which respond with locomotor deficits, a lower survival, and/or reduced reproduction (Desneux et al. 2004; Ceuppens et al. 2015; Charreton et al. 2015). Such negative consequences of exposure to agrochemical pollutants may become even worse if insects are already challenged by other environmental factors such as parasites.

Studies which considered the effects of parasites and pesticides on (non-target) organisms indicate increased detrimental effects on fitness-related traits when these challenges were tested in combination (Coors and De Meester 2008; Fauser-Misslin et al. 2014; Botías et al. 2021). For example, bumblebee colonies (*Bombus terrestris*) infected with a microsporidium gut parasite and exposed to different insecticides showed reduced growth (Fauser-Misslin et al. 2014). Survival of water flea populations (*Daphnia magna*) was reduced by synergistic negative actions of parasitism and exposure to the insecticide 1-naphthyl methylcarbamate (Coors and De Meester 2008). To our knowledge, studies investigating the consequences of a gregarine infection on terrestrial arthropods in combination with insecticide exposure are lacking. However, detrimental effects on fitness can occur as responses of gregarine-infected hosts exposed to suboptimal food conditions (Harry 1967; Zuk 1987b). These effects may be similar to responses of gregarine-infected hosts to sublethal insecticide exposure, because such exposure leads to a suppressed food intake, as shown, for example, in adults of the mustard leaf beetle *Phaedon cochleariae* (Coleoptera: Chrysomelidae) (Wolz et al. 2021).

Larvae and adults of *P. cochleariae* feed on different Brassicaceae plants and can be infected with a prevalent gregarine species that colonises the gut (Müller et al. 2017a). The gregarine load of the larvae has been shown to relate to the host plant species and thus food quality (Müller et al. 2017a). The habitat of the leaf beetle includes both agricultural and natural areas. In the latter, it can be considered as a non-target organism (Müller and Müller 2016) that faces traces of insecticides either through misapplication or drift from insecticide spray dust. Exposure of adults to sublethal concentrations of the pyrethroid λ-cyhalothrin resulted in impaired food consumption, delayed development, and reduced reproduction in the exposed as well as the subsequent unexposed generation (Müller et al. 2017b, 2019a; Wolz et al. 2021). In larvae, sublethal λ-cyhalothrin exposure caused detrimental effects on different life history traits and altered interspecific interactions with potential predators (Müller et al. 2019b).

To understand whether the presence/absence of a biotic challenge can modulate insect responses to an anthropogenic environmental stressor, we investigated the effects of gregarine infection and larval exposure to a sublethal concentration of λ-cyhalothrin. Therefore, we studied the effects of both factors individually and in combination on the food consumption and several life history traits, using *P. cochleariae* as a suitable model. Moreover, we assessed the infection load with gregarines in larvae and adult beetles. Based on the assumption that gut endoparasites deprive nutrients from the host, we expected an increased food consumption by gregarine-infected larvae. We predicted that consumption is also enhanced after insecticide exposure, as a suppressed food intake during insecticide exposure may be compensated. Furthermore, we hypothesised that an infection of *P. cochleariae* with gregarines is not harmful, but in combination with a sublethal insecticide exposure, life history traits are impaired, leading to a reduced fitness. Finally, we expected that detrimental insecticide exposure leads to a higher number of gregarines in the host.

## Materials and methods

### Study organism and rearing

A rearing of *P. cochleariae* was maintained for several generations at Bielefeld University under constant climatic conditions (20 °C, 16 h: 8 h light: dark, 70% r.h.). Adult beetles were mixed randomly for mating and groups of 100–200 individuals kept in boxes (20 × 20 × 6.5 cm) covered with gauze lids. Every year, the rearing population was replenished with individuals collected in the field (51° 51′ 21″ N, 8° 41′ 37″ E). Larvae and beetles were supplied with leaves of non-flowering 8–10-week-old cabbage plants (*Brassica rapa* L. spp. *pekinensis*), which were cultivated in pots in a greenhouse (20 °C, 16 h: 8 h light: dark, 70% r.h.). For the experiment, only middle-aged leaves were offered, and larvae were provided with greenhouse-grown cabbage, while adults received cabbage bought from an organic store due to plant shortage.

### General experimental set-up and measurements of larval food consumption and life history traits

A full-factorial design was set up to test the influences of gregarine infection and sublethal insecticide exposure on larval consumption and life history of *P. cochleariae*. Three-week-old adults (about 200 individuals) were provided with cabbage leaves and their offspring was subsequently used for the experiment. Female beetles bite little cavities in the leaf surface, lay individual eggs in these cavities, and cover the eggs with secretion (Müller and Rosenberger 2006). Infection of larvae of *P. cochleariae* with gregarines occurs via the uptake of spores excreted by infected conspecifics (Müller, unpublished). Faecal remains can also cover the eggs. Thus, after 24 h of oviposition time, eggs were carefully removed from the leaves and the secretion and potential faeces removed with a paintbrush and tap water. Eggs were placed on fresh cabbage leaves and randomly distributed over two rearing boxes, one assigned to the uninfected gregarine treatment (G −: *N* = 325) and the other to the gregarine-infection treatment (G + : *N* = 320). Hatching larvae (G −: *N* = 195, G + : *N* = 204) were supplied with the respective food sources for 4 days as described below (experimental infection of *P. cochleariae* larvae with gregarines) to ensure a G − or G + treatment.

At the fourth day after hatching, the larvae were divided into groups of 5–10 larvae in large Petri dishes (9 cm diameter) lined with filter paper and provided with cabbage leaf pieces (3 × 4 cm). From day 5 on, half of both G − and G + larvae were assigned to one of the two insecticide treatment groups, either receiving no insecticide (I −) or receiving λ-cyhalothrin-treated leaf discs (I +) for 48 h (procedure see below), resulting in four treatment groups (G – I −: *N* = 50, G − I + : *N* = 113, G + I −: *N* = 60, G + I + : *N* = 130). Much higher numbers of I + animals were set up to account for a lower survival of insecticide-exposed individuals. From day 7 after hatching, all individuals were provided with untreated cabbage leaves (ESM Fig. S1).

To investigate the effects of the different treatments on consumption, the amount of leaf mass consumed within 24 h by each larva was measured. At day 9 after hatching, two larvae were randomly selected from each Petri dish. Each larva was weighed (microbalance, ME36S, Sartorius AG, Göttingen, Germany), placed in a small Petri dish (5.5 cm diameter) lined with moistened filter paper and offered a leaf disc (24 mm diameter) of known mass (balance, LA120S-OCE, Sartorius AG, Göttingen, Germany). After 24 h, the larvae were weighed again and the remaining leaf discs were scanned (Samsung ProXpress SL-M3375FD, Schwalbach/Taunus, Germany; resolution: 600 dpi). The remaining leaf area was determined with ImageJ (v 1.52a) and used to calculate the consumed leaf mass [initial leaf mass × (initial leaf disc area − remaining leaf area)/initial leaf disc area]. Afterwards, these larvae remained in their individual Petri dishes and were used for counting of gregarines (see below).

To test the effects of the different treatments on life history traits, pupae were individually placed in small Petri dishes lined with filter paper and the time from larval hatching until adult eclosion was noted. The body mass of adult individuals was measured 24 h after adult eclosion and the sex of the individuals determined. Adults were kept individually in small Petri dishes and supplied with cabbage leaves. Eight days after adult eclosion females were mated with males of the same treatment group, placing each male to one female for 24 h. After mating, the pairs were separated again, and the number of eggs laid within the subsequent 4 days per female was counted. The hatching rate was determined by counting the number of hatched larvae relative to the number of eggs laid. Moreover, the survival of all individuals kept under the four treatment regimes was noted until day 10 after adult eclosion. The experiment was ended when adults were 15 days old, while adults can live up to 3 months (Bogdanov-Katjkov 1923).

### Experimental infection of *P. cochleariae* larvae with gregarines

To ensure larval infection with gregarines (G + treatment), leaves covered with faeces were taken that had been placed in the boxes of the insect rearing stock for 24 h. Random examinations of the rearing stock showed that all tested beetles were infected with gregarines and that infectious spores were present in their faeces. Larvae of the G − treatment were offered leaves that had been kept for 24 h in a box without conspecifics and were damaged by regular cuttings to imitate feeding and thus provide leaves of comparable quality to those provided to the G + group. Such leaves were offered to hatching larvae of the respective treatment groups and replaced every 2 days until larvae were 4 days old. Microscopy of larvae and adults (see below) confirmed that all dissected individuals of the G − treatment (*N* = 40 larvae, *N* = 46 adults) were not infected, while all individuals of the G + treatment were successfully infected with gregarines (*N* = 40 larvae, *N* = 73 adults).

### Preparation of sublethal λ-cyhalothrin concentration and insecticide exposure treatment

The pyrethroid λ-cyhalothrin was chosen as contact insecticide, because it is widely used in agriculture, including fields with crops of Brassicaceae (Soderlund et al. 2002). A sublethal concentration was prepared from the insecticide LAMBDA WG, which contains 5% of the active toxin λ-cyhalothrin (Syngenta 2016). The insecticide was dissolved in methanol (HPLC-grade, VWR International GmbH, Darmstadt, Germany) and centrifuged, and the supernatant used to prepare a 0.6 mg/L λ-cyhalothrin stock solution. This concentration was considered as sublethal, as less than 50% of the larvae died during the exposure period (following the definition by de França et al. (2017)). In an agricultural system, this concentration is approximately less than half of the application recommended by the supplier (Syngenta 2016) and may thus occur in areas close to treated fields as residue. The experimental larvae were fed on days 5 and 6 after hatching with cabbage leaf discs (24 mm diameter) treated with 76.8 µL methanol (I −) or λ-cyhalothrin solution (I +). The surfaces of the leaf discs were evenly covered with the respective solutions using a pipette and leaf discs were kept under a fume hood until evaporation of the solvent (at least 20 min) before offering them to the larvae. Depending on the number of larvae, two-to-four leaf discs were offered per Petri dish to provide food ad libitum, and after one day, the leaf discs were exchanged by discs of the respective treatments for another day.

### Counting of gregarines

Subgroups of both larvae and adults of *P. cochleariae* were examined to test whether the number of gregarines depends on the different treatments and developmental stage of the host. At day 12 after larval hatching, a subset of larvae of the different treatments (two larvae per Petri dish, used for consumption assay, *N* = 20 larvae per treatment group) was taken and individuals were frozen (− 20 °C). Likewise, adult males and females of each treatment group (G − I −: *N* = 9 females, *N* = 12 males; G − I + : *N* = 12 females, *N* = 13 males; G + I −: *N* = 12 females, *N* = 15 males; G + I + : *N* = 26 females, *N* = 20 males) were randomly selected and frozen at day 15 after adult eclosion. Afterwards, individuals were thawed, the midgut dissected, spread on a microscope slide in 25 µL sodium phosphate buffer (0.1 M, pH = 7.2), and the number of gregarines (likely as trophozoites and gamonts) counted under a light microscope at 200–400 times magnification (ZEISS Axiophot, Carl Zeiss Microscopy GmbH, Jena, Germany) (ESM Fig. S2).

### Statistical analyses

All statistical analyses were performed in R version 3.6.3 (R Core Team 2020) with RStudio version 1.2.5033 (RStudio Team 2019). Data were analysed using linear models (lm), generalised linear models (glm) (package MASS; Venables and Ripley 2002), and cox proportional hazard models (package survival; Therneau 2020). Model assumptions for lms and glms (normal distribution and homoscedasticity of residuals) and cox proportional hazard models (proportional hazard and influential observations) were checked using diagnostic plots. Stepwise backward model selection was applied to obtain the minimal adequate models. Based on F test or Chi-square test results (package MASS; Venables and Ripley 2002), non-significant (*P*-value > 0.05) interaction terms and/or predictors were excluded from the models.

The effects of the predictors gregarine treatment and insecticide treatment as well as their interaction on development time (from larval hatching to adult eclosion) were tested using a glm (Poisson distribution, identity link function). The influences of the predictors larval body mass, gregarine treatment, and insecticide treatment and their interaction on the food consumption of larvae were analysed using a lm. The effects of the predictors gregarine treatment, insecticide treatment, as well as their interaction on the adult body mass were analysed separately for males and females using lm. Glms were performed to investigate the effects of gregarine treatment, insecticide treatment and their interaction on the number of eggs laid by adult females (Poisson distribution, log link function), and the hatching rate of larvae [binomial family (success/failure of hatching), logit link function]. To investigate the effects of insecticide treatment on the gregarine load in the larval stage, a glm (Poisson distribution, identity link function) was calculated. For the gregarine load in adults, a glm (Poisson distribution, identity link function) with insecticide treatment and sex as predictors was performed. The influences of the predictors gregarine treatment, insecticide treatment, and their interaction on survival probability were tested using a cox model, followed by pairwise log-rank post hoc tests. Survival data were plotted using Kaplan–Meier curves (package survival; Therneau 2020).

## Results

### Effects of gregarine infection and sublethal λ-cyhalothrin exposure on larval food consumption and life history traits

**Fig. 1:**
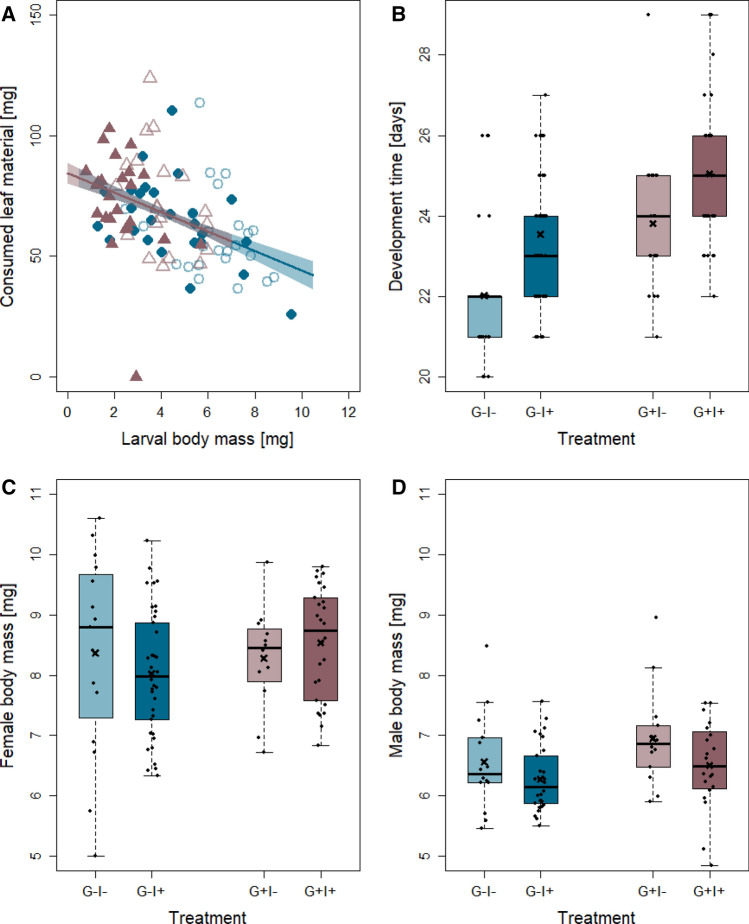
Effects of gregarine (G) treatment and insecticide (I) treatment on larval food consumption (fresh weight) (**A**), development time (**B**), and adult body mass of females (**C**) and males (**D**) of *Phaedon cochleariae*. Overlaid lines for the food consumption represent model predictions of linear model with associated SE as polygons around the lines and raw data points (open circles: G – I −, filled circles: G − I + , open triangles: G + I −, and filled triangles: G + I +). Box plots are overlaid with raw data and show the medians (horizontal lines), means (crosses), 25th and 75th percentiles, and 1.5 × lower and upper quartiles (whiskers)

The food consumption of larvae was neither influenced by gregarine treatment nor insecticide treatment, but was significantly affected by the larval body mass (lm: *df* = 1, *F* = 20.2, *P* < 0.001; G − I −: *N* = 24, G − I + : *N* = 26, G + I −: *N* = 20, G + I + : *N* = 24). Heavier larvae consumed less leaf material than lighter larvae (Fig. 1A).

The developmental time was significantly affected by the gregarine treatment (Table 1). Uninfected larvae developed on average about 1.5 (I +) to 2 (I −) days faster compared to the gregarine-infected insects (Fig. 1B).

**Table 1:** Effects of gregarine treatment and insecticide treatment on performance traits of *Phaedon cochleariae*

Trait	*df*	*F/X*^2^	*P*
Development			
Gregarine treatment (G)	1	3.9	**0.046**
Female body mass			
Gregarine treatment (G)	1	1.9	0.172
Male body mass			
Insecticide treatment (I)	1	5.5	**0.021**
Number of eggs			
Insecticide treatment (I)	1	8.9	** < 0.01**
Hatching rate			
Insecticide treatment (I)	1	36.5	** < 0.001**

Results are based on the minimal adequate model (only predictors are shown that remained in each model). For linear models, (lm) *F*-values and for generalised linear models (glm) Wald *X*^2^-values are shown. Significant *P* values (*P* < 0.05) are indicated in bold. Number of replicates per treatment group between 12 and 47

Neither the body mass of females nor that of males was affected by the gregarine treatment (Table 1, Fig. 1C, D). However, the body mass of the males was significantly influenced by insecticide exposure, with I + beetles being lighter than I − individuals.

**Fig. 2:**
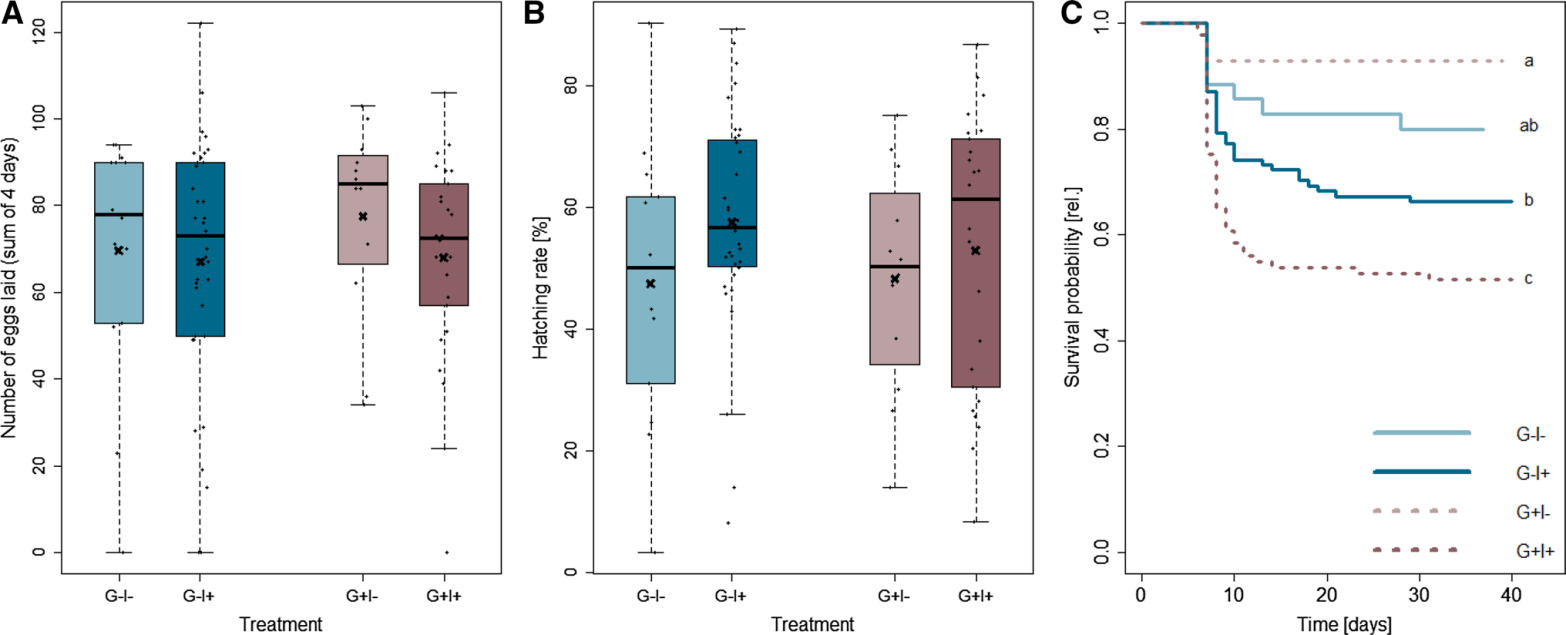
Effects of gregarine (G) treatment and insecticide (I) treatment on number of eggs laid (**A**), hatching success (**B**) and survival probability (**C**) of *Phaedon cochleariae*. Box plots are overlaid with raw data, and show the medians (horizontal lines), means (crosses), 25th and 75th percentiles, and 1.5 × lower and upper quartiles (whiskers). The survival rates are illustrated as Kaplan–Meier survival curves. The survival was scored from larval hatching until day 10 after adult eclosion (initial G – I −: *N* = 35, G − I + : *N* = 101, G + I −: *N* = 28, G + I + : *N* = 89) per treatment group. Different lower case letters indicate significant differences between treatment groups

Female reproduction was not affected by gregarine treatment, but insecticide treatment had a significant impact on the number of eggs laid and the hatching rate (Table 1). When not exposed to insecticides, females laid on average between 4% (G −) and 14% (G +) more eggs compared to insecticide-exposed females (Fig. 2A). In contrast, the hatching success of unexposed females was on average 18% lower in the uninfected group (G −) and 3% in the gregarine-infected group (G +) compared to the insecticide-exposed females (Fig. 2B).

The survival probability of *P. cochleariae* ranged between 50 and 90% at 10 days after adult eclosion (Fig. 2C). The survival probability was influenced by the interaction of gregarine treatment and insecticide treatment (cox model: *X*^2^ = 4.4, *P* = 0.036, G + I −: *N* = 35, G − I + : *N* = 101, G + I −: *N* = 28, G + I + : *N* = 89). Individuals with gregarine infection and no insecticide exposure (G + I−) had the highest survival, whereas gregarine infection and insecticide exposure (G + I +) resulted in the lowest survival probability.

**Fig. 3:**
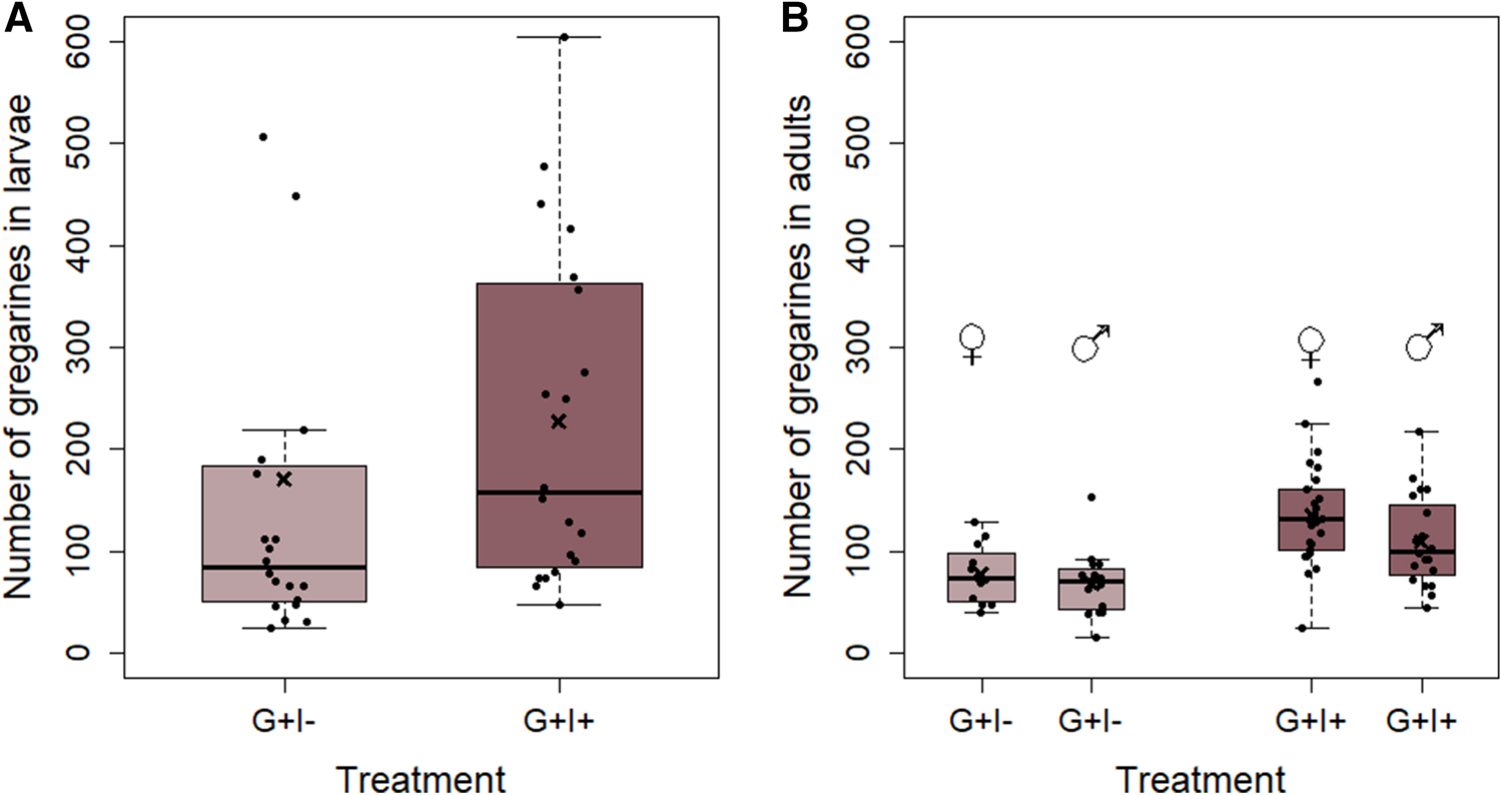
Effects of insecticide (I) treatment on number of gregarines (likely trophozoites and gamonts) in larvae (**A**) and adult females and males (**B**) of *Phaedon cochleariae*. Box plots are overlaid with raw data, and show the medians (horizontal lines), means (crosses), 25th and 75th percentiles, and 1.5 × lower and upper quartiles (whiskers). No gregarines were found in G − animals

### Effects of sublethal λ-cyhalothrin exposure on gregarine load

No gregarines were found in the G − individuals (*N* = 40 larvae, *N* = 46 adults). In the gregarine-infected animals, significantly less gregarines were found in unexposed than in insecticide-exposed larvae (glm: *df* = 1, *X*^2^ = 157.8, *P* < 0.001, G + I −: *N* = 20, G + I + : *N* = 20) (Fig. 3A). In adults, the number of gregarines was likewise significantly affected by the insecticide treatment (glm: *df* = 1, *X*^2^ = 417.31, *P* < 0.001, G − I + : *N* = 12 females, *N* = 15 males; G + I + : *N* = 26 females, *N* = 20 males) and in addition by the sex of the beetles (glm: *df* = 1, *X*^2^ = 66.72, *P* < 0.001). As in the larvae, unexposed adult beetles showed less gregarines, but overall females were more heavily infected by gregarines than males (Fig. 3B).

## Discussion

Our study revealed that the two challenges, infection with gregarines and larval exposure to sublethal λ-cyhalothrin concentrations, had distinct effects on *P. cochleariae* when tested individually or in combination. Against our expectation, larval food consumption was neither significantly affected by gregarine infection nor by sublethal insecticide exposure, but highly dependent of the larval body mass. It has been suggested that an infection of the gut with gregarines leads to a depletion of nutrients (Gigliolli et al. 2016), and therefore, we assumed an enhanced food consumption to compensate for such depletion. However, in other insects, such as the European earwig (*Forficula auricularia*), gregarine infection did not alter the food intake of the host (Arcila and Meunier 2020). In contrast, a gregarine infection for 20 days significantly reduced food intake in the tobacco grasshopper (*Atractomorpha crenulata*), which consequently had a lower body mass (Johny et al. 2000). Thus, effects on consumption may be species-specific and/or depend on the timing of events. In our experiment with *P. cochleariae*, the time span between the gregarine infection and the food consumption assay was relatively short (5 days in between). At a later time point, when gregarines developed further in the larval midgut, insect larvae may become more deprived of nutrients.

Larvae of *P. cochleariae* can regulate their food consumption depending on the quality of their food (Tremmel and Müller 2013) and adults increased their food consumption several days after a 2-week exposure to a sublethal λ-cyhalothrin concentration (Wolz et al. 2021), presumably to compensate for the reduced food intake during exposure. In the present study, we exposed the larvae for a relatively short time (2 days), which probably caused a less drastic reduction in food intake, and thus, no compensatory food consumption was observed. Nevertheless, food consumption of these larvae was highly dependent on their body mass, with lighter larvae consuming more than heavier larvae. Interestingly, larvae of *P. cochleariae* with gregarine infection were lighter than larvae without gregarine infection (see ESM S3). A higher consumption of lighter individuals may be necessary to catch up and reach a similar larval body mass as uninfected individuals before entering metamorphosis.

An infection with gregarines resulted in a delayed development, which is likewise known for other terrestrial arthropods (Zuk 1987a; Thomas and Rudolf 2010; Gigliolli et al. 2016). This negative effect of gregarines on larval development was independent of insecticide exposure, in contrast to our initial hypothesis that gregarine infection only in combination with another challenge, i.e., sublethal insecticide exposure, leads to a reduced fitness. An extended development time of the larvae, which are usually less mobile than the adults, may enhance their predation risk (Häggström and Larsson 1995) and reduce the number of generations per year (Cole 1954). A slower development is often associated with a restriction in resources during early life development (Metcalfe and Monaghan 2001). In addition to nutrient deprivation, an accumulation of gregarines can hinder the passage of food or lead to morphological (e.g., damage of epithelial cells in midgut) and physiological alterations (e.g., impaired hormone regulation), interfering with the host development (Sanders and Poinar 1973; Gigliolli et al. 2015). Overall, gregarines may cause metabolic costs, which ultimately lead to a delayed development, which was also reflected in the reduced larval body mass of *P. cochleariae* larvae at day 9 (see ESM S3).

Other life history traits than larval development were not affected by the gregarine infection in the present study, but in part by the insecticide exposure. Only males, but not females, showed a reduced body mass, but females produced less eggs when exposed as larvae to λ-cyhalothrin. In general, responses to environmental stress in larval stages can affect allocation patterns to later life history traits like adult body mass, size, or reproduction (Boggs 2009; Dmitriew and Rowe 2011). The negative consequences of the larval insecticide exposure on male body mass and egg deposition may result from resource- and energy-demanding metabolic detoxification processes (Dawkar et al. 2013; Hafeez et al. 2020). A low male body mass can negatively affect reproduction, as it may influence mating success (Candolin 2005; Murphy and Krupke 2011). For females, exposure to pyrethroids in larval stages is known to lead to a reduced fat content in adult damselflies (*Coenagrion scitulum*) (Dinh et al. 2016), which may result in less energy allocation towards egg production. Whether the fat content of females of *P. cochleariae* is affected by insecticide exposure of the larvae remains to be tested.

Despite the reduction in the number of eggs laid, the hatching rate of larvae from these eggs was higher in individuals that had experienced larval insecticide exposure. Laying fewer eggs but providing them with more resources may reflect a trade-off to compensate for limited resources, which has been found, for example, in soil mites (*Sancassania berlesei*) and European earwigs (Benton et al. 2005; Koch and Meunier 2014). Alternatively, the higher hatching rate may point to a hormetic effect, i.e., stimulatory effects of low levels of insecticide on reproduction (Guedes et al. 2010). Hormetic effects have been, for example, found in adults of the Colorado potato beetle (*Leptinotarsa decemlineata*), which had a higher body mass and were more likely to survive if the larvae had been exposed to a pyrethroid (Margus et al. 2019). However, it is questionable how persistent this positive effect on the hatching success of *P. cochleariae* is, as we did not measure the reproductive output of the females across lifetime, but only over a limited time period. Finally, not all eggs may have been fertilized, which may have contributed to reduced hatching rates.

In line with our expectation, the combination of gregarine infection and insecticide exposure resulted in the lowest survival probability of *P. cochleariae*, indicating that potential detrimental effects of a gregarine infection sometimes only become apparent when insects are exposed to additional environmental challenges. In contrast, gregarine-infected individuals without insecticide exposure showed the highest survival probability, which points in the direction of a mutualistic relationship between host and gregarine. Therefore, the classification of gregarines as parasites is not fully justified for the leaf beetle and shows that gregarines should be more neutrally considered as endosymbionts (Rueckert et al. 2019). The switch of a symbiotic relationship between host and “parasite” from mutualistic to parasitic can be triggered by extreme environmental conditions, such as starvation (Harry 1967; Zuk 1987a; Moran et al. 2020). The outcome of such interactions is moreover highly species-specific. For example, starvation in combination with a high gregarine infection led to a higher survival probability in the European earwig (Arcila and Meunier 2020), but a lower longevity of field crickets (Zuk 1987b). Because the counting of gregarines is invasive, we could not compare the gregarine load for dead individuals with individuals still living. However, our data may indicate a negative relationship at the larval stage, where mortality is peaking.

Furthermore, our results revealed that insecticide exposure increased the number of gregarines found in both larvae and adults of *P. cochleariae*. This finding may be attributed to weakening of the host by the insecticide, for example, through resource-demanding detoxification processes that can lower the immune protection (Rivero et al. 2010). A higher gut parasite load by *Nosema* spp. has been detected in honeybees exposed to imidacloprid (Pettis et al. 2012), and likewise, more tapeworms (*Hymenolepis diminuta*) were found in mealworm beetles (*Tenebrio molitor*) following pyrethroid exposure (Dhakal et al. 2020) compared to insects without insecticide exposure. Other studies suggest that the gregarine-infection level plays a crucial role in the effect of gregarines on their hosts, with higher loads causing obviously negative effects (Sulaiman 1992; Field and Michiels 2005).

In adults of *P. cochleariae*, the number of gregarines was lower than in the larvae. It is unknown how and in which stage gregarines persist across the metamorphosis in this beetle species. In the red flour beetle (*Tribolium castaneum*), the gregarines are likely excreted during metamorphosis (Thomas and Rudolf 2010; Critchlow et al. 2019). A reinfection of adults of this species resulted in a significantly reduced gregarine load, if larvae had been already infected (Thomas and Rudolf 2010), providing evidence for an immune-priming process in gregarine-infected individuals. Furthermore, we detected a higher gregarine-infection load of females compared to males of *P. cochleariae*. Sex-specific differences in the gregarine prevalence have been reported for earwigs and odonate species, with biases towards males (Córdoba-Aguilar and Munguía-Steyer 2013; Arcila and Meunier 2020) or females (Bunker et al. 2013), but also no sex-differences have been found (Ilvonen et al. 2016). The differences in prevalence may be attributed to differences in behaviour between the sexes. For instance, sex-specific food intake may lead to an uptake of more spores in the sex that consumes more food (Hecker et al. 2002). Alternatively, a higher infection load in females compared to males of the leaf beetle may be due to their size dimorphism; females are heavier compared to males and may therefore host a higher number of gregarines by potentially providing more resources. Irrespective of the causes, we likely can exclude an additional burden by gregarines on females, as their reproduction was not impaired by gregarines.

In conclusion, our study revealed that both naturally prevalent gregarines and sublethal pyrethroid exposure, at least in the concentration tested here, impact different fitness traits of a leaf beetle species individually or in combination. The interactive negative effects of both factors on survival indicate a very complex relationship between challengers and the host. Under field conditions, combined effects may be enhanced in stressful natural environments, in which individuals likely face numerous challenges. Similar effects may be expected for other (non-target) insects, although the influence of gregarines is mostly host-specific. Therefore, to counteract the global decline of insects, the potential of natural parasite infections in modulating insect responses to anthropogenic and non-anthropogenic environmental factors should be considered for ecological risk assessment and their effects on ecosystems.

## Supplementary Information

Below is the link to the electronic supplementary material.

Supplementary file1 (PDF 409 KB)

## Data Availability

All data and code from this study are available via the Dryad Digital Repository (https://doi.org/10.5061/dryad.k6djh9w6h).
