# Supplementary material for: Gregarines modulate insect responses to sublethal insecticide residues
Source: Oecologia. 2021 Dec 1;198(1):255–65. doi: 10.1007/s00442-021-05086-4 (PMC8803800; doi:10.1007/s00442-021-05086-4)
Supplement: Supplementary file 1 — Supplementary file1 (PDF 409 KB) [file 442_2021_5086_MOESM1_ESM.pdf]

Oecologia

Electronic Supplementary Material (ESM)

Article title: Gregarines modulate insect responses to sublethal insecticide residues

Authors: Marina Wolz, Alia Schrader, Eileen Whitelaw, Caroline Müller

The following Supporting Information is available for this article:

Figure S1: Experimental design

Figure S2: Dissection of *Phaedon cochleariae* beetles and light microscope images of gregarines

S3: Effects of gregarine treatment and insecticide treatment on larval body mass

Figure S1: Experimental design. Eggs of *Phaedon cochleariae* were collected and after larval hatching, larvae were either assigned to the uninfected treatment group (G-) or to the gregarine-infected treatment group (G+). Gregarine infection was ensured by feeding the larvae with food, which contained faecal remainings of infected conspecifics over 4 days. From day 6 after larval hatching, the larvae either received no insecticide (I-) or sublethal λ-cyhalothrin treated food (I+) for 48 hours, resulting in four treatment groups (G-I-, G-I+, G+I-, G+I+). At day 9 after larval hatching, larvae were weighed and a food consumption assay (over 24 hours) was performed. At day 12, larvae from each treatment group were dissected to count the number of gregarines. After pupation and adult eclosion, beetles were weighed and sexed. From day 8 to 12 after adult eclosion, female beetles were mated (at least for 24 hours) and afterwards the number of laid eggs from each female was calculated over 4 days as well as the hatching success of these eggs. The survival probability was calculated until day 10 after adult eclosion and at day 15 adult beetles were dissected to count the number of gregarines.

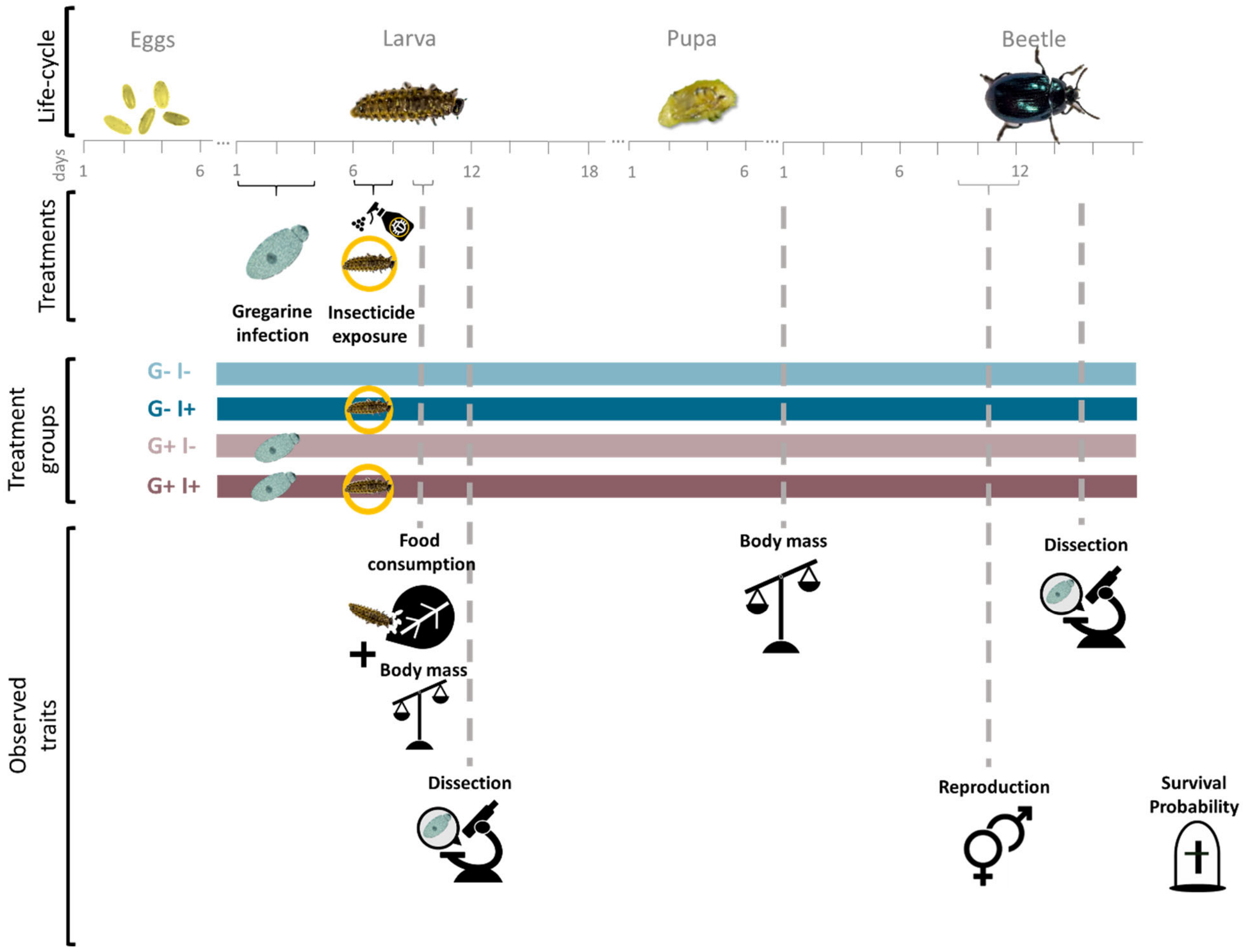

Figure S2: Dissection of *Phaedon cochleariae* beetle (A: left beetle, right gut) and light microscope images of single gregarines (presumably trophozoit stage) (B) and two associated gregarines (presumably gamont stage) (C) at 200- or 400-fold magnification.

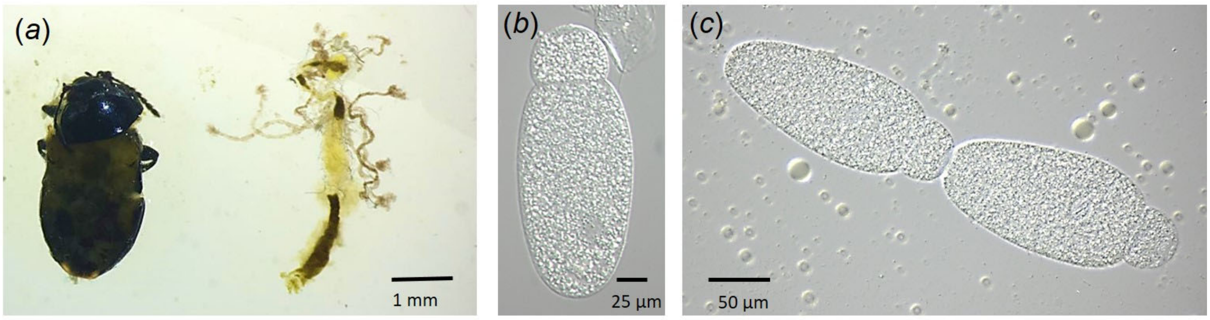

S3: Effects of gregarine treatment and insecticide treatment on larval body mass

To investigate the effects of the gregarine infection and insecticide exposure on larval body mass at day 9 after hatching, two larvae were randomly selected from each Petri dish. Each larva was weighed individually using a microbalance (Microbalance, ME36S, Sartorius AG, Göttingen, Germany). The effects of gregarine treatment and insecticide treamtent as well as their interaction on the larvae body mass were assesed using a linear model. Results are based on the minimal adequate model (non-significant interaction terms were excluded).

The larval body mass was significantly affected by the gregarine treatment (*df* = 1, *F* = 52.2, *P* < 0.001) and the insecticide treatment (*df* = 1, *F* = 38.2, *P* < 0.001). The body mass of non-infected larvae (G-) was higher compared to the gregarine-infected larvae (G+) and the body mass was reduced with insecticide exposure (I-) (Fig. S3).

Fig. S3:

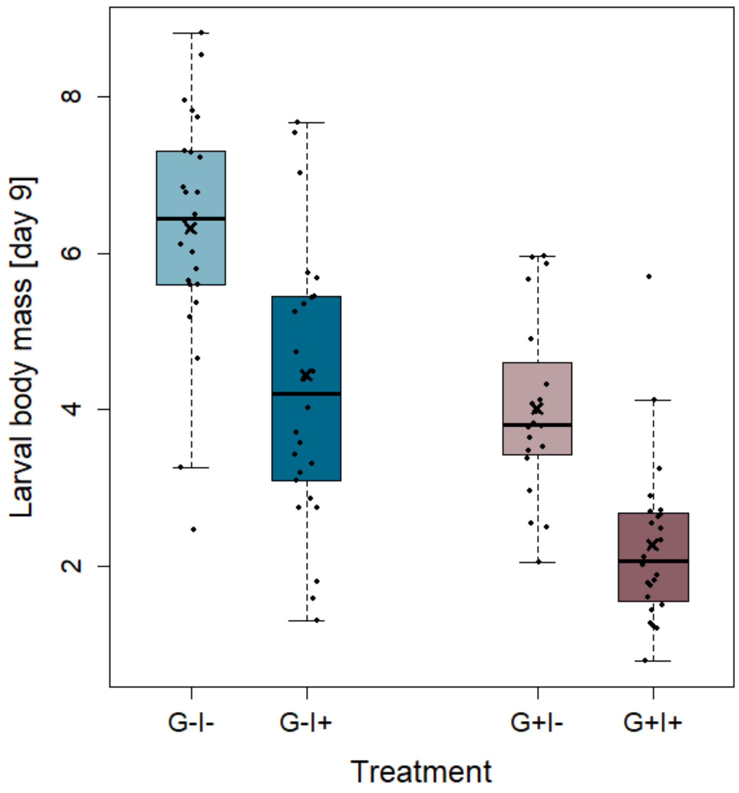

Fig. S3: Effects of gregarine (G) treatment and insecticide (I) treatment on larval body mass. Box plots are overlaid with raw data and show the medians (horizontal lines), means (crosses), 25th and 75th percentiles and 1.5 × lower and upper quartiles (whiskers).
